# Current Status of Probiotics as Supplements in the Prevention and Treatment of Infectious Diseases

**DOI:** 10.3389/fcimb.2022.789063

**Published:** 2022-03-14

**Authors:** Xinquan Li, Qiang Wang, Xiafen Hu, Wanxin Liu

**Affiliations:** Institute of Infection, Immunology and Tumor Microenvironment, Hubei Province Key Laboratory of Occupational Hazard Identification and Control, Medical College, Wuhan University of Science and Technology, Wuhan, China

**Keywords:** probiotics, infectious disease, microbiota, antibiotics, microecology

## Abstract

Probiotics play an important role against infectious pathogens *via* their effects on the epithelium, the production of antimicrobial compounds, and competitive exclusion. Administration of probiotic supplements may reduce the risk of infectious diseases and the use of antibiotics, hence contributing to a reduction or a delay of the development of multi-resistant bacteria. Infection is a constant concern for people who experience recurrent infections, and antibiotic treatment usually fails due to antibiotic resistance. Therefore, an infection can lead to severe illness and hospitalization if left untreated. A growing number of studies have demonstrated promising results for a variety of probiotic strains used to prevent or treat acute and recurrent infectious diseases, but additional standardized clinical research is needed.

## Introduction

Homeostasis is a dynamic equilibrium that is maintained in body tissues and organs. Environmental factors, such as exposure to infectious pathogens, can constantly interact with human commensal bacteria and the immune system, both of which work together to shape an individual’s health conditions. Metabolites produced by the microbiota and their components are essential for maintaining immune homeostasis. They are also vital for affecting a host’s vulnerability to immune-mediated diseases and disorders. There is progressive recognition that microbiota-generated derivatives play a crucial role in human physiology, which has profound effects on the immune system. From these observations, one concept that can lend support is that mammals are holobionts; they rely on both host and microbial genomes, i.e., the hologenome, for optimal function (Rooks and Garrett, 2016).

The consistent high occurrence of antimicrobial-resistant chronic, emerging infectious diseases remains a considerable global health threat. There is increasing awareness of the essential role that the microbiota plays in health (Shahmanesh et al., 2020). Colonization of microbial communities induces the “colonization resistance” of an array of molecular mechanisms that affect physiological functions. Microbe–microbe and microbe–host interactions are involved in these colonization-regulating activities (Leshem et al., 2020). Probiotics are live microorganisms that, when ingested in sufficient amounts, would confer health benefits to the host (Rastelli et al., 2019) and influence the physiology of the host through their metabolic activity, hence are effective therapeutic tools in diverse diseases (Falcinelli et al., 2018). In particular, they play a significant role in infectious diseases (**Table 1**), the beneficial effects of which are elaborated in the following section.

**Table 1:** Probiotics play a role in the human body.

Reference	Disease location	Subject	Outcome
Szajewska and Mrukowicz (2001); Sartor (2005); Zhang et al. (2015)	Gastrointestinal tract	Patients with diarrhea and *Helicobacter pylori*	Duration of diarrhea ↓
			*H. pylori* eradication rates ↑
Hao et al. (2011); Weng et al. (2017)	Respiratory tract	URTi and VAP patients	Incidence of URTi and VAP↓
Hanson et al. (2016)	Vagina	Women with urinary tract infection	Improvement of vaginal microecology
Barker et al. (2020)	Mammary gland	Perinatal parturient	Incidence of mastitis ↓
			Improvement of mammary microecology
Wang et al. (2021)	Respiratory tract	Potential infected individuals from URTi	Administration of *S. thermophilus* ENT-K12 for 30 days and establishment of the oropharyngeal flora to protect frontline medical staff
			Incidence of URTi, antibiotics, duration of symptoms, absent from work ↓

↓ denotes a decrease; ↑ denotes an increase.

URTi, upper respiratory tract infection; VAP, ventilator-associated pneumonia.

## Protective Mechanisms of Probiotics Against Infectious Diseases

There is extensive consideration that probiotics are essential dietary components that can reduce the risk of infectious diseases due to their functions in mediating the immune responses in epithelial cells, which are associated with systemic immune functions and the epithelial mucosa. Probiotics are “live microorganisms that, when administered in adequate amounts, confer a health benefit to the host” (Hill et al., 2014). A prebiotic is defined as “a substrate that is selectively utilized by host microorganisms, conferring health benefits” (Gibson et al., 2017). The human symbiotic flora, which gradually evolved into an advanced ecosystem that can produce unique metabolites acting on other cells, is a rich source of potential antimicrobials (Liu et al., 2018). Probiotics have already shown great achievement in the manufacture of wine and yogurt before being used in medicine. When it was discovered that yogurt can treat diarrhea, probiotics officially played a role in the medical arena (Ozen and Dinleyici, 2015). The mechanisms of how probiotics help protect a host from infections include strengthening of the epithelial barrier, increasing adhesion to the intestinal mucosa, inhibiting pathogen adhesion, competitively rejecting pathogenic microbiota, synthesizing antibacterial substances, modifying toxins or toxin receptors, and stimulating nonspecific and specific immune responses to pathogens (Bermudez-Brito et al., 2012).

Probiotic-derived factors can interact with intestinal epithelial cells. Probiotic fimbriae are the key to the adhesion to the human mucosa and intestinal epithelium (Simon et al., 2021). In addition, they also promote the elimination of pathogens in competition with other pathogens (Segers and Lebeer, 2014). In a model of human fetal ileum organs, *Lacticaseibacillus rhamnosus* was found to weaken the inflammatory response caused by *Salmonella* through subunits of pili (Ganguli et al., 2015). The involvement of *L. rhamnosus* pili in promoting intestinal crypt cell proliferation has also been demonstrated in animal models (Ardita et al., 2014). When probiotics adhere to human intestinal epithelial cells, they will affect the intestinal microecology and, thus, the human body in various aspects. For example, the p40 protein produced by *L. rhamnosus* can activate the epidermal growth factor (EGF) receptor in intestinal epithelial cells (Yan and Polk, 2020). This protects the intestinal epithelium from tumor necrosis factor (TNF)-induced injury (Yan et al., 2013). In a rat gastrectomy model, high-dose probiotics (*Lactiplantibacillus plantarum*, *L. rhamnosus*, and *Lacticaseibacillus acidophilus*) can downregulate the levels of inflammatory proteins caused by the activation of Toll-like receptor 4 (TLR4)/nuclear factor kappa B (NF-κB) signal pathway (Zheng et al., 2021). Also, the structural components of probiotics themselves can regulate the number of immune cells and the secretion of cytokines, such as polysaccharide A (PSA) in *Bacteroides fragilis* that modulates the innate immune system of the host through TLR2 in dendritic cells, thereby increasing the production of regulatory T cells and interleukin 10 (IL-10) (Round et al., 2011). Moreover, the short-chain fatty acids (SCFAs) produced by probiotics can alter the acid–base environment of the intestine, thereby inhibiting the growth of pathogens (van Zyl et al., 2020). Regarding the immune response, SCFAs can reduce intestinal permeability, circulating endotoxin, and inflammation and oxidative stress (Kim et al., 2018). Bile salt hydrolases, products of probiotics, can also promote the secretion of satiety hormones (Lebeer et al., 2018).

The hydrogen peroxide produced by symbiotic and probiotic bacteria is an important antibacterial mechanism in the vaginal environment. Studies have shown that the hydrogen peroxide-producing *Lactobacillus F117* and *Paralactobacillus F28* can inhibit the growth of *Staphylococcus aureus in vitro*, and women carrying H_2_O_2_-producing lactic acid bacteria are less likely to suffer from bacterial vaginosis (BV) (van Zyl et al., 2020). The mechanism may be that the colonization of lactic acid bacteria-producing H_2_O_2_ can regulate the levels of IL-1β, SLPI, and HBD2 (Mitchell et al., 2015).

Oral administration of probiotics also has an impact on the immune response within the respiratory tract, with the interaction of the bacterial structure or metabolites with receptors [such as Toll-like receptors (TLRs)] on host epithelial and immune cells driving their direct role in immune function, such that probiotics can engage and activate TLRs, thereby activating NF-κB and interferon regulatory factors (IRFs) in immune cells, which are critical for antiviral defense. It has been shown *in vitro* that probiotics may stimulate the innate immune pathways similar to those stimulated by respiratory viruses, thereby modulating virus-induced immune responses (Lehtoranta et al., 2020). The mechanical nature of probiotics also allows them to perform competitive inhibition with pathogens by adhesion competition for binding sites, such that probiotics can form a protective layer after adhesion to epithelial cells or mucus surfaces, thereby blocking the contact between pathogens and host cells (Popova et al., 2012).

Derivatives of probiotics can also transmit signals through the central nervous system, such as the production of γ-aminobutyric acid (GABA) through tryptophan derivatives (Janik et al., 2016). In a chronic lead exposure rat model, probiotics could normalize the morphology of dendritic spines in the hippocampi of small trees after chronic lead exposure and reverse the level of H3K27me3 in the hippocampus, which could alleviate pro-neurotoxicity. Meanwhile, probiotics could also alleviate the peripheral inflammation induced by lead by affecting the level of IL-6 (Zheng et al., 2021).

Overall, there have been numerous clinical cohorts and animal models demonstrating that the administration of probiotics exerts benefits in various organs and systems in the human body. A large number of studies have focused on the alterations of the gut microecology and the interaction between the gut and other body parts in order to demonstrate the mechanisms of probiotics. More studies are needed to help improve our understandings of the protective mechanisms at play between infectious diseases and other microecological systems, such as the interaction between oropharyngeal microecology and respiratory tract infectious diseases.

## Probiotics Protect Host From Diarrhea and *Helicobacter pylori* Infection

Regarding infectious diarrhea, acute diarrhea in infants and children is defined as three or more loose or watery stool in 24 h. Randomized, double-blind, placebo-controlled trials on probiotics for the treatment or prevention of acute diarrhea demonstrated that the use of probiotics significantly reduced the risk of diarrhea lasting more than 3 days compared with placebo, particularly in rotaviral gastroenteritis (Szajewska and Mrukowicz, 2001). As inferred from recent reviews, specific probiotics have shown efficacy in acute viral gastroenteritis and antibiotic-associated diarrhea (such as *Clostridioides difficile* toxin-induced diarrhea). A review showed that probiotics may reduce *C. difficile* infections by 50% in high-risk groups (Mills et al., 2018). In *in vitro* experiments on *C. difficile* infections, the serine protease M-protease produced by *Bacillus clausii* protected mammalian cell lines from *C. difficile* toxin (Ripert et al., 2016), and the *Lacticaseibacillus reuteri* strain 17938 was shown to directly inhibit *C. difficile in vitro* (Spinler et al., 2017). In addition, animal models also provided proof that *Lacticaseibacillus plantarum* and the prebiotic xylitol could inhibit the ability of spores of *C. difficile* to germinate, and the mortality rate of mice consuming *L. plantarum* and the prebiotic xylitol before administration of *C. difficile* decreased from 44% to 22% (Rätsep et al., 2017). For antibiotic-associated diarrhea and *C. difficile* infections, the combination of *Lactobacillus* and *Bifidobacterium* species decreased *C. difficile* toxin-positive diarrhea to 2.9% in elderly patients on antibiotics for various indications, while the diarrhea rate in the placebo group was 7.3% (Plummer et al., 2004). In another large prospective study on 1- to 5-year-olds treated with antibiotics, *Saccharomyces boulardii* decreased the rate of diarrhea from 18.9% to 5.7% (La Rosa et al., 2003). The combination of probiotic (*Lacticaseibacillus sporogenes*) and prebiotic (fructooligosaccharides) reduced the incidence of diarrhea from 71% to 38% in 120 children treated with antibiotics with clinical indications and decreased the duration of diarrhea from 1.6 to 0.7 days (Sartor, 2005).

Rotavirus is responsible for one-third of the cases of severe diarrhea in children younger than 5 years worldwide (Lopez-Santamarina et al., 2021). A meta-analysis of rotavirus diarrhea in children showed that probiotic use in children (not limited to *Lacticaseibacillus lactis*, *Lacticaseibacillus paracasei*, *Bifidobacterium longum*, *Bifidobacterium infantis*, *Enterococcus faecalis*, and *S. boulardii*) can not only effectively prevent diarrhea caused by rotavirus but also reduce the symptoms of fever and vomiting after diarrhea occurrence (Yang et al., 2019). Furthermore, it was demonstrated in a rotavirus infection model on neonatal Lewis rats that *Bifidobacterium breve* M-16V, *L. acidophilus* NCFM, and *Lacticaseibacillus helveticus R0052* can reduce the incidence and severity of diarrhea (Azagra-Boronat et al., 2020). Regarding the mechanism of action of probiotics on rotavirus diarrhea, cellular experiments have demonstrated that probiotics can increase the production of interferon gamma (IFN-γ) in cells in response to rotavirus infection, and they also increased the expression of the *CXCL10* gene (Ishizuka et al., 2016). In animal models, probiotics have been shown to induce the mucosal production of protective factors, decrease the concentrations of pro-inflammatory cytokines, and increase antibodies specific to rotavirus (Preidis et al., 2012; Kawahara et al., 2017).

Randomized controlled experiments investigating the standard treatment of *Helicobacter pylori* eradication *versus H. pylori* combined with probiotic therapy showed that the overall eradication rate in the probiotic group was approximately 10% higher than that in the control group. Furthermore, the incidence of adverse events in the probiotic group decreased by nearly 15% compared to that in the control group. It was also found that the concomitant use of probiotics and standard therapy increased the *H. pylori* eradication rate by approximately 13% and reduced adverse events by approximately 41% (Zhang et al., 2015).

As a typical consideration, the gut microbiota is the first line of defense against enteric pathogenic microorganisms. Regular consumption of probiotic foods and/or supplements is essential to maintaining the symbiotic balance of the gut microbes and the host. However, selection of different probiotics in different therapeutic regimens for gastrointestinal (GI) infectious diseases should be based on the mechanisms of action of the different probiotics. Overall, the role of probiotics, whether in maintaining balance in the healthy state of the gut or aiding in recovery in states of infection, cannot be overstated.

## Probiotics Play a Role in the Prevention and Treatment of Respiratory Infections

The oropharyngeal microbiota is diverse, with more than 700 different bacterial species found in the tongue, teeth, gingiva, inner cheek, palate, and tonsils. Although saliva does not contain salivary indigenous bacteria, it contains bacteria shed from other areas of the oral cavity. More than 20% of oral bacteria are *Streptococcus* spp., the most predominant genus in the oral cavity (Evans, 2016).

Whether an individual is tolerant or susceptible to upper respiratory tract infection (URTi) is associated with the mucosal immunity of their upper respiratory tract (URT), which depends to a great extent on the type and abundance of bacteria in the oral cavity, which in turn are determined, predominantly, by one’s age, health status, nutritional status, lifestyle (i.e., tobacco and alcohol use along with the quality of oral hygiene), and geographic residence (Thibeault et al., 2021).

### Probiotic Preparations Reduce the Odds of Respiratory Tract Infections and Antibiotic Use by Regulating Oropharyngeal Microflora

Probiotics were found to be advantageous for reducing both the episodes of acute respiratory tract infections and antibiotic use (Hao et al., 2011). A systematic review also showed that the oropharyngeal probiotic *Streptococcus salivarius* K12 that colonizes the oropharyngeal mucosa may be useful in reducing the occurrence and/or severity of acute otitis media and secretory otitis media in children, as middle ear infections typically occur after the spread of nasopharyngeal bacteria through the Eustachian tube (Zupancic et al., 2017). Another systematic review reported that *S. salivarius* K12 prophylaxis significantly lowered the incidence of streptococcal pharyngitis (Wilcox et al., 2019). Considering that early supplementation with prebiotics or probiotics reduces the risk of developing virus-associated respiratory infections in the first year of life in a cohort of preterm infants, Finnish researchers conducted a randomized, double-blind, placebo-controlled trial on 94 preterm infants and found that the administration of prebiotics or probiotics significantly lowered the incidence of respiratory tract infection (RTi); the incidence of rhinovirus-induced seizures (accounting for 80% of all RTi attacks) was also significantly reduced (Luoto et al., 2014).

For young children attending kindergarten who usually suffer from seasonal RTi, a randomized study showed that those who had just started attending kindergarten and who received *S. salivarius* K12 for 90 days had a significant reduction in the incidence of infections, including tonsillopharyngitis, tracheitis, rhinitis, laryngitis, and otitis media, and a significant reduction in the use of antipyretics, antibiotics, and the number of days absent from preschool during treatment and within 6 months of follow-up (Tetyana and Olha, 2021).

Several studies have shown that the administration of *Streptococcus thermophilus* K12 is a promising strategy for the prevention of streptococcal and viral pharyngotonsillitis, and its high safety profile was also confirmed (**Table 2**).

**Table 2:** Application of *Streptococcus salivarius* K12 for respiratory tract infections.

Reference	Disease	Subject	Outcome
Di Pierro et al. (2016)	Streptococcal pharyngo-tonsillitis	Children attending the first year of kindergarten	During the 6 months of treatment: streptococcal pharyngo-tonsillitis ↓ (67%)
	Acute otitis media		Acute otitis media ↓ (45%)
			During the 3-month follow-up: streptococcal pharyngo-tonsillitis ↓ (42%)
			Acute otitis media ↓ (67%)
Francesco et al. (2016)	Pharyngotonsillitis	Pediatric subjects with non-recurrent streptococcal infection	After the administration of *Streptococcus salivarius* K12 for two trimesters out of 12 months: pharyngo-tonsillitis ↓ (90%)
	Acute otitis media		
			Acute otitis media ↓ (70%)
Karpova and Karpycheva (2019)	Adenoiditis exacerbation	Children presenting with clinical and anamnestic signs of chronic adenoiditis	Administration of *Streptococcus salivarius* K12 for 30 days in combination with nasal douche: adenoiditis exacerbation ↓ (44%)
	Acute sinusitis		After 3 months: acute sinusitis ↓ (73%)
	Acute otitis media		Acute otitis media ↓ (62%)
			Requirement for medication therapy ↓

Ilchenko et al. (2020)	Chronic tonsillitis	Children suffering from chronic tonsillitis	Cases of tonsillar hypertrophy and low-grade fever ↓
			Normalization of blood counts
			Isolations of *Staphylococcus aureus* in the diagnostic titer, bacterial count of group A beta-hemolytic streptococcus, alone and together with *S. aureus* ↓
			Acute respiratory viral infections ↓
Di Pierro et al. (2015)	Otitis media	Children with a recent history of recurrent acute otitis media and with unilateral or bilateral fluid in the middle ear for at least 2 months	After treatment with *Streptococcus salivarius* K12 for 90 days: occurrence and/or severity of secretory otitis media ↓
			Acute otitis media ↓

↓ denotes a decrease; ↑ denotes an increase.

An alternative to colonizing probiotics is the use of nasal or oral sprays to alter the aggressiveness of nasopharyngeal pathogenic microbiota or their ability to affect the overall health of the host (Benninger et al., 2011). The pathogen levels can be reduced by recolonization with commensal bacteria, thereby reducing the likelihood of new respiratory infections and the recurrence rate of pharyngeal tonsillitis and acute otitis media (Falck et al., 1999; Roos et al., 2001).

A growing number of studies have demonstrated the protective effects of probiotics against common respiratory infections such as the common cold and influenza, and these studies supported the idea that probiotic supplementation can help improve childhood and adult immunity to the common cold, which can reduce the incidence, duration, and symptoms of cold. As mentioned above, probiotics play an important role in reducing severe respiratory infections. In summary, current studies have focused on the preventive effects of probiotics against respiratory infections, and supplementation of ingested probiotics in healthy adults has shown improvement in immune function; immunoassays for common cold infection should be enhanced. However, the mechanisms of how probiotics exert their effects on the immune response to influenza, as well as their duration, dose, and type of effectiveness, require further study. This is why probiotics are not widely used for respiratory infections at present. Once the mechanisms by which they exert their effects in the respiratory tract have been explicitly elucidated, their use in the clinic may not be limited only to the prevention of infections but also as an adjunct in the treatment of infections.

The number of bacterial strains resistant to antibiotics has dramatically increased during the past decades. Bacteriocin, which is produced by commensal bacteria, could be exploited as a backup to antibiotic treatment. It was highlighted that bacteriocin cocktail released by the commercialized strain *S. salivarius* K12, which inhibits respiratory pathogens such as *Streptococcus pyogenes*, has been clinically proven to reduce the antibiotic absorption doses *via* reducing the recurrent episodes of streptococcal pharyngitis, tonsillitis, or otitis (Hols et al., 2019).

### Probiotics Against Ventilator-Associated Pneumonia in Critically Ill Patients

It is worth mentioning that infection in critically ill patients during their stay in the intensive care unit (ICU) remains a major challenge, and there have been numerous clinical trials evaluating the efficacy of probiotics in preventing infectious complications, especially ventilator-associated pneumonia (VAP). VAP in mechanically ventilated patients contributes to morbidity and mortality, but the role of probiotics in the prevention of VAP is controversial. A multinational ICU study of 14,414 patients in 1,265 ICUs in 75 countries and territories showed that patients with infectious diseases had more than twice the mortality rate as non-infected patients, whereas 64% of the infections in infected patients were of the respiratory tract (Jean-Louis, 2009). The preventive effect of probiotics on VAP infection in mechanically ventilated patients has been comprehensively evaluated in a meta-analysis and trial sequential analysis including 13 randomized controlled trials (RCTs) with a total of 1,969 subjects. Overall, probiotics reduced the incidence of VAP, but there was no significant difference in overall mortality, ICU mortality, hospital mortality, length of ICU stay, length of hospital stay, or duration of mechanical ventilation (Weng et al., 2017). Dysbiosis is a characteristic of critical illness leading to the overgrowth of potentially pathogenic bacteria, which can cause high susceptibility to nosocomial infections. Other systematic reviews and meta-analyses that included 30 RCTs involving 2,972 participants assessed whether probiotics are associated with a significant reduction in infection. There was a significantly lower incidence of VAP in the group using probiotics. In addition, subgroup analyses exploring the effects of probiotics on other clinical outcomes demonstrated that the prophylactic effect of a mixture of probiotics and symbiotic alone on infection was most pronounced in critically ill patients (Manzanares et al., 2016). Probiotics have shown promise to be able to reduce infections, and VAP in critical illness is no exception. Nevertheless, the findings from current studies have been inconsistent due to clinical heterogeneity, and the clinical application of probiotics is still quite conservative. Thus, further clinical trials that can demonstrate the benefits of probiotics are still needed.

## Probiotics Improve Female Genitourinary Tract Infections by Regulating Vaginal Flora

Recurrent urinary tract infections (UTIs) burden a great number of women worldwide. Probiotics, especially *Lacticaseibacillus*, have been considered to prevent UTI. *Lacticaseibacillus* plays a major role in the urogenital tract flora of healthy premenopausal women, thus creating the concept that the recovery of urogenital tract pathogens from the urinary tract by lactic acid bacteria can prevent UTIs. Most of the clinical trials using specific strains conducted in women with UTIs or prophylaxis use against uropathogens already had encouraging findings.

### Both Oral Probiotics and Vaginal Suppositories Improve the Vaginal Flora

Restoration of the urogenital flora can be confirmed by the detection of probiotics or the increase of *Lacticaseibacillus* in the vaginal environment. Vaginal instillation of the probiotics *L. rhamnosus* GR-1 and *Limosilactobacillus fermentum* RC-14 in healthy women without urogenital infections can be detected from cultures of vaginal swabs of all subjects after 3 days. Less than half of women had healthy vaginal flora at study entry, although none had symptomatic urogenital infection, oral administration of *L. rhamnosus* GR-1 and *L. fermentum* RC-14 resulted in more than half of the women with abnormal vaginal flora reverting to normal within 28 days (Reid et al., 2001; Cadieux et al., 2002).

### Probiotics as Adjunctive Therapy to Antibiotics Decrease the Risk of Recurrent UTIs

Numerous clinical trials have shown the significant impacts of probiotics in preventing UTI recurrence, as probiotics have demonstrated the ability to restore vaginal flora dysbiosis in patients with BV. This provides a rationale for combining probiotics with antibiotics for the treatment of BV (**Table 3**).

**Table 3:** Use of *Lacticaseibacillus* in urinary tract infections (UTIs).

Reference	Experimental approach	Probiotic strain	Method of administration	Outcome
Reid et al. (2003)	Randomized, double-blind, placebo-controlled trial	*L. rhamnosus GR-1*	Oral administration	*Lacticaseibacillus* ↑
		*L. fermentum RC-14*		Yeast and coliforms ↓
				Vaginal health and vaginal itchiness or odor had been improved.
Reid et al. (1995)	Randomized, double-blind, placebo-controlled trial	*L. rhamnosus GR-1*	Intravaginal suppository	UTI rate ↓ (73% in 1 year)
		*L. fermentum B-54*		
Macklaim et al. (2015)	16S rRNA gene sequencing	*Lactobacillus reuteri RC-14*	Oral capsule	Relative abundance of indigenous *Lactobacillus iners* or *Lactobacillus crispatus* ↑
		*L. rhamnosus GR-1* (with tinidazole)		
Homayouni et al. (2014)	Randomized controlled trials	*L. acidophilus*	Oral consumption	Normalization of the vaginal flora
		*L. rhamnosus GR-1*		Cure of the existing infection
		*L. fermentum RC-14*		Prevention of the recurrence of BV

↓ denotes a decrease; ↑ denotes an increase.

BV, bacterial vaginosis.

### Probiotics Reduce the Risk of Preterm Delivery by Reducing the Onset of Bacterial Vaginitis

A systematic review further indicated that, although the studies focusing on BV, UTIs, vulvovaginal candidiasis, and human papillomavirus (HPV) were heterogeneous in terms of their design, intervention, and outcomes, the effectiveness of probiotic intervention in the treatment and prevention of bacterial vaginitis has been elaborated. In addition, probiotics play a role in preventing *Candida* infection and UTI recurrence. Moreover, probiotics help improve the clearance of HPV, while showing a high safety profile (Hanson et al., 2016). Various gynecologic and obstetric conditions are clinically associated with BV, which is found in about one in five pregnant women considered to be at higher risk of preterm birth. Several studies showed that when pregnant women suffering from BV consumed yoghurt that contains live *L. acidophilus*, a significant increase of positive vaginal cultures with *L. acidophilus* and a significant reduction in the number of episodes of BV occurred (Shalev, 2002). There is ample rationale for the use of probiotics in pregnant women. Certain *Lacticaseibacillus* can colonize the vagina without side effects after oral and vaginal administration, kill and replace pathogens, reduce the risk of preterm birth by interfering with the inflammatory cascade, and promote embryonic development through their potential in regulating the levels of lipids and cytokines (Reid and Bocking, 2003).

Probiotics can replace unhealthy microbiota and occupy specific adhesion sites on the urothelial surface, thus maintaining an acidic vaginal environment through the production of hydrogen peroxide and lactic acid. Vaginal or oral administration enables probiotics to colonize the vagina. Although there are studies with varying results, most of them suggested a role for probiotics in the prevention or treatment of vaginal infections, and no adverse effects have been reported. This is also the reason probiotics can be directly applied as an adjunctive therapy for vaginal infectious diseases. Furthermore, specific strains that currently produce marked effects on vaginal health have also been found. Therefore, the daily consumption of probiotic products containing certain strains is recommended to improve women’s health.

## Probiotics Prevent Mastitis by Reducing the Content of Staphylococci in the Mammary Glands

Human mastitis, including acute mastitis and subacute mastitis, is defined as breast infection characterized by various local symptoms. In some cases, mastitis also has systemic symptoms. In fact, mastitis is a process of dysbiosis of the mammary glands, which is mainly caused by *S. aureus*. *S. aureus* would proliferate and produce toxins, causing strong inflammation once in the mammary gland, and an intense local inflammatory response follows. The mammary gland is highly vascularized throughout lactation, and once infected with mastitis, toxins produced by *S. aureus* are rapidly absorbed and enter the bloodstream, altering the host cytokine pattern; systemic influenza-like symptoms. Coagulase-negative staphylococci (CNS) and viridans streptococci are normal species of the mammary microbiota. During lactation, they line the epithelium of the mammary ducts to form a thin biofilm that allows the normal flow of milk. Overgrowth of CNS and viridans streptococci may be favored by different factors, thus leading to subacute or subclinical mastitis. Since toxins such as those produced by *S. aureus* are not produced by viridans streptococci, subacute and subclinical mastitis do not have systemic flu-like symptoms; however, they are able to form a thick biofilm within the ducts that inflames the mammary epithelium and forces milk through increasingly narrower lumens, and the pressure on the inflamed epithelium can cause stinging pain with breast cramps and burning sensations that further block milk flow (Fernández et al., 2014).

Lactation mastitis commonly occurs in breastfeeding women, which is a painful inflammation that can cause decreased immunity, lowered resistance to infection, and the early cessation of breastfeeding, which in turn may lead to poorer maternal and neonatal outcomes. As few as 1 in 5 breastfeeding women will be affected by mastitis, which mostly begins 6–8 weeks postpartum. Antibiotics are common treatments for mastitis, but they have not been shown to be effective prophylactics. An RCT including 625 participants who had just undergone production showed a 51% reduction in the incidence of mastitis at 16 weeks with oral *L. fermentum* CECT5716 during lactation. In addition, the load of *Staphylococcus* spp., a common cause of mastitis, at the end of the intervention was significantly lower in breastfeeding group compared to the control group (Bond et al., 2017; Hurtado et al., 2017). A scoping review of RCTs indicated that probiotics may have utility in the treatment and prevention of lactational mastitis (Barker et al., 2020).

Furthermore, the concept of the entero-mammary route has proven that live bacteria from the maternal GI tract can be transferred to the mammary gland *via* the mesenteric lymph node network. Oral administration of certain probiotic strains during pregnancy and lactation can modulate the flora of mesenteric lymph nodes and mammary gland microbiota to improve mammary gland health and, ultimately, improve maternal and neonatal health during lactation (Lassen et al., 2015; Treven et al., 2015; Sakwinska and Bosco, 2019).

Since probiotics can improve breast health through the entero-mammary route, regular and quantitative use of probiotics in pregnant women preparing to breastfeed should be recommended in order to achieve a higher breastfeeding rate. Relevant probiotic species should also be developed more, as probiotics are expected not only to prevent mastitis but also, more greatly, to serve as adjunctive therapy at the onset of mastitis to improve maternal breastfeeding duration.

## Can Probiotics Flatten the Curve of the COVID-19 Pandemic?

As acute lung injury-mediated lung dysbiosis has been demonstrated to be associated with blood-mediated modulation of the gut microbiota, coronavirus disease 2019 (COVID-19) may induce lung microbiota disruption, thus driving the imbalance of the gut microbiota (Olaimat et al., 2020). By investigating the lung tissue of deceased COVID-19 patients, it was found that lung microbiome dysbiosis in severe COVID-19 patients was characterized by enrichment of bacteria that are primarily responsible for the highest rates of multidrug resistance and mortality; therefore, continuous monitoring of the lower respiratory tract microbiota is needed for prompt and personalized treatment (Batista et al., 2021). The occupational health impact of respiratory infectious diseases is costly to the economy and the healthcare system. A scoping review of occupational health stated that probiotics may be a therapeutic tool for URTIs by improving the immune system and reducing the days off work. Practically no adverse effects in the populations tested, including frontline health workers, were seen (Picó-Monllor et al., 2021). The aforementioned studies have demonstrated that the use of probiotics and prebiotics could be a preventive nutritional strategy in managing respiratory infections such as COVID-19, as well as their anti-inflammatory effects against the main signs and symptoms associated with COVID-19.

### The Upper Respiratory Tract May Be a Hotbed of SARS-CoV-2 Infection in the Lower Respiratory Tract

It is known that most cases of COVID-19 transmission occurred *via* person-to-person respiratory droplets and from environmental surfaces to the hands and then to the nose and mouth. Both pathways allow the virus to reach, as a first step, the URT, from where it can spread to the lungs. The establishment of a first line of defense in the URT and the oral microbiota to prevent URTi, including COVID-19, could be a promising strategy.

Human angiotensin-converting enzyme 2 (ACE2) is remarkably abundant on the membrane of lung alveolar epithelial cells and enterocytes, in particular in buccal cells, where it was expressed in 0.52% of buccal cells, with a predominance of tongue epithelial cells, demonstrating that there is interaction between severe acute respiratory syndrome coronavirus 2 (SARS-CoV-2) and the microorganisms in the lungs or oral cavity (Viana et al., 2020). SARS-CoV-2 utilizes the ACE2 receptor to bind to the cells in the lung, where it can cause severe, and possibly fatal, pneumonia (Di Pierro, 2020). ACE2 plays an important role in the balance of the intestinal flora. Once it is shed, it has a substantially increased potential for gut microbial dysbiosis, which may be responsible for the adverse outcomes in elderly COVID-19 patients. This mechanism also ultimately explains the increased frequency of metabolic syndrome, ICU admissions, and mortality in these patients (Viana et al., 2020). The crucial role of ACE2 has been tested through several therapeutic approaches. Indian researchers searched for probiotic produced bacteriocins that could block the ACE2 site by calculating molecular docking using bacteriocin metabolites produced by different probiotic species (Anwar et al., 2020). The treatment of dysbiosis could turn out to be helpful for immunomodulation, and probiotics may support patients by inhibiting the ACE2 receptor, i.e., the passage of the virus into the cell, and may also be effective in suppressing the immune response caused by the pro-inflammatory cytokine cascade (Hawryłkowicz et al., 2021).

According to a meta-transcriptome sequencing study on bronchoalveolar lavage fluid samples, the microbiota of patients infected with SARS-CoV-2 were similar to those in CAP (virus-like community-acquired pneumonia), with either pathogen levels, oral and URT symbiotic microorganisms, being increased (Shen et al., 2020). In a large number of coinfected cases, the opportunistic pathogenic pathogen originating from the oral cavity was identified in the bronchoalveolar lavage fluid of a patient with novel coronavirus pneumonia, suggesting that the oral cavity may be a warm bed for the pathogen in patients with novel coronavirus pneumonia coinfections. Saliva appears to be a favorable clinical specimen, while the viral load of SARS-CoV-2 was reported to be highest in oropharyngeal saliva samples in the first week after the onset of symptoms, suggesting that SARS-CoV-2 has a high affinity for the oral epithelium (Bao et al., 2020).

### Probiotics Help Establish the Oropharyngeal Microflora Against SARS-CoV-2

Poor oral hygiene is considered to be a major ecological pressure that steers complex microbial communities in the mouth into dysbiosis. Available data indicate that the oral cavity may be an active site of infection and may influence SARS-CoV-2 infection (Xiang et al., 2021). A metagenomic analysis of the oropharyngeal microbiota in patients with COVID-19 revealed that variations in the oropharyngeal microbiome in COVID-19 may be used as a biomarker for dysbiosis of the pulmonary microbiome, which is associated with lung co-infections in COVID-19, to help guide antibiotic treatment (Ma et al., 2021). Characterization of the oropharyngeal microbiome in recovered COVID-19 patients showed increased abundance of the butyrate-producing *Fusobacterium* that could promote intestinal mucosal barrier repair, which can contribute to recovery from COVID-19, and a decreased abundance of the lipopolysaccharide (LPS)-producing opportunistic pathogen *Leptotrichia*, which could induce systemic pro-inflammatory-promoting COVID-19 development (Gao et al., 2021). Notably, oral dysbiosis correlated with symptom severity and increased local inflammation, while a decreased mucosal sIgA (secretory immunoglobulin A) response was observed in more severely symptomatic COVID-19 patients, suggesting that local immune response is important in the early control of virus infection and is influenced by the oral microbiome profile (Soffritti et al., 2021). It was reported that the use of the oropharyngeal probiotic *S. thermophilus* ENT-K-12, in a slow-dissolving lozenge form (twice a day), was able to create a stable URT microbiota, significantly reducing the incidence of certain respiratory tract infections [22/95 (23.2%) in the control group *vs*. 8/98 (8.2%) in the probiotic group, *p* < 0.004] among frontline physicians and nurses attending to COVID-19 patients in a hospital setting (Wang et al., 2021). A preliminary study has shown that prophylactic administration of *S. salivarius* K12 in children may reduce the risk of SARS-CoV-2 infection. In this study, a certain number of the children had close contact with family or classmates who tested positive for COVID-19. Both the presence of typical COVID-19 symptoms and the incidence of SARS-CoV-2-specific positive antigen tests were seen to a greater extent in untreated children (Di Pierro and Colombo, 2021).

These findings may be important in allowing new therapeutic opportunities addressed to establish a more balanced oropharyngeal microflora that reduces an individual’s susceptibility to SARS-CoV-2 infections, induces immune responses associated with reducing viral replication, or modulates inflammation to prevent the development of severe COVID-19 symptoms.

In conclusion, probiotics can provide protection from both URTi and gut dysbiosis caused by viral infections and enhance the immunity of individuals *via* an activated immune system. Probiotics can also downregulate the inflammatory reaction induced by SARS-CoV-2 infection, known as a cytokine storm, which was directly correlated with viral pneumonia and serious complications of SARS-CoV-2 infections, consequently flattening the curve of the COVID-19 pandemic. The administration of probiotics to healthcare workers and practitioners in the context of the COVID-19 pandemic could be a safe and effective prevention approach, along with proper protective equipment. However, the beneficial effects of probiotics are strain-specific; hence, more solid clinical data and a scientifically proven pathophysiological link between systemic inflammatory response and the lungs and gut in the progression of COVID-19 are needed.

## Future Considerations

In this era of increasing bacterial resistance, it is undoubtedly important to develop therapies that can replace antibiotics and that are harmless to humans. The selection of specific probiotics for infection sites after an infection is one of the most encouraging treatment options for the prevention of common acute infections in the post-antibiotic era (De Francesco et al., 2010; Jakobsson et al., 2010; Zielnik-Jurkiewicz and Bielicka, 2015). Antimicrobial peptides produced by probiotics are a new trend in existing research, known as bacteriocin, which may be used to substitute antibiotics or to reduce the emergence of resistant strains. Fortunately, many bacteria colonized in the human body can be mobilized to produce bacteriocin and prevent infection. This provides a promising prospect for the treatment of multidrug-resistant infections (Cotter et al., 2013; Barbour et al., 2016; Hols et al., 2019; King et al., 2019).

Infections caused by multidrug-resistant bacteria are a serious clinical problem, and bacteriocins that can significantly reduce multidrug-resistant strains may have narrow- or broad-spectrum activities, which deserve serious consideration as alternatives to antibiotics. The Swedish government has been taking action in 2000, with the development of national targets for outpatient prescription quantity, identification of quality indicators based on treatment recommendations, and local feedback to prescribing physicians and educational activities. Currently, the level of antibiotic use and resistance in Sweden are now the lowest in the human and animal domains in European Union (EU) countries. Between 1992 and 2016, the number of prescriptions in outpatient care and primary healthcare decreased by 43%, while among children aged 0–4 years, these decreased by 73%. It is worth noting that the sales of antibiotics for respiratory tract infections have declined (Mölstad et al., 2017).

Probiotics, in particular, have great potential in the context of today’s increasing threat of antibiotic overuse and the prevalence of antibiotic-resistant microorganisms. A comprehensive reduction in antibiotic use is a widely adopted public health goal, and suitable probiotics that reduce infections still need to be discovered. Research on probiotics to restore dysfunctional bacteria has produced mixed results, while studies aimed at selecting scientific strains may still provide reliable solutions to replacing antibiotics in the future.

## Author Contributions

QW organized the writing of this review. XL, XH, and WL bisected the remaining task of writing. All authors contributed to the article and approved the submitted version.

## Funding

The authors are cordially thankful to 2020 General Planning Fund Project for Humanities and Social Sciences of the Ministry of Education, China (Project No. 20YJA880053); Key research project of philosophy and social sciences of Hubei Provincial Department of Education in 2020 (Project No. 20D026); WUST National Defence Pre-research Foundation, China (Project No. GF202003) and Hubei Province Key Laboratory of Occupational Hazard Identification and Control, Wuhan University of Science and Technology (Project No. OHIC2020G05) for their financial support to conduct this project successfully.

## Conflict of Interest

The authors declare that the research was conducted in the absence of any commercial or financial relationships that could be construed as a potential conflict of interest.

## Publisher’s Note

All claims expressed in this article are solely those of the authors and do not necessarily represent those of their affiliated organizations, or those of the publisher, the editors and the reviewers. Any product that may be evaluated in this article, or claim that may be made by its manufacturer, is not guaranteed or endorsed by the publisher.
